# Tomato MicroRNAs and Their Functions

**DOI:** 10.3390/ijms231911979

**Published:** 2022-10-09

**Authors:** Tzahi Arazi, Jackson Khedia

**Affiliations:** Institute of Plant Sciences, Agricultural Research Organization-Volcani Institute, HaMaccabbim Road 68, P.O. Box 15159, Rishon LeZion 7505101, Israel

**Keywords:** tomato, miRNA, microRNA, Atlas, CRISPR, ShortStack, annotation, fruit, Solanum

## Abstract

MicroRNAs (miRNAs) define an essential class of non-coding small RNAs that function as posttranscriptional modulators of gene expression. They are coded by *MIR* genes, several hundreds of which exist in the genomes of Arabidopsis and rice model plants. The functional analysis of Arabidopsis and rice miRNAs indicate that their miRNAs regulate a wide range of processes including development, reproduction, metabolism, and stress. Tomato serves as a major model crop for the study of fleshy fruit development and ripening but until recently, information on the identity of its *MIR* genes and their coded miRNAs was limited and occasionally contradictory. As a result, the majority of tomato miRNAs remained uncharacterized. Recently, a comprehensive annotation of tomato *MIR* genes has been carried out by several labs and us. In this review, we curate and organize the resulting partially overlapping *MIR* annotations into an exhaustive and non-redundant atlas of tomato *MIR* genes. There are 538 candidate and validated *MIR* genes in the atlas, of which, 169, 18, and 351 code for highly conserved, *Solanaceae*-specific, and tomato-specific miRNAs, respectively. Furthermore, a critical review of functional studies on tomato miRNAs is presented, highlighting validated and possible functions, creating a useful resource for future tomato miRNA research.

## 1. Introduction

Plant genomes code for various non-coding small RNAs that play important roles in genetic and epigenetic silencing [1]. According to their size, biogenesis, and mode of action, plant small RNAs have been classified into several types [2]. MicroRNAs (miRNAs) are an intensively studied class of plant small RNAs that have been demonstrated to be involved in a broad range of biological processes including reproduction, differentiation, development, signaling, metabolism, and the response to biotic and abiotic stresses [3,4,5,6]. MiRNAs are typically 20- to 22-nucleotides (nt) long (canonical miRNAs) and rarely 24 nt long (long miRNAs) [7,8]. As opposed to most small interfering RNAs (siRNAs), which are processed from double-stranded RNAs, miRNAs are processed from single-stranded stem-loop RNA precursors [2]. A plant genome typically contains a few hundred *MICRORNA* (*MIR*) genes, which are grouped into families based on the sequence similarity of their coded miRNAs. In addition, miRNAs show variable degrees of conservation across species boundaries [9,10]. Evolutionarily conserved miRNAs are usually coded by multigene families, whereas the less conserved lineage- and species-specific miRNAs are coded by small families or even by a single *MIR* gene [11].

The transcription of a *MIR* gene produces a poly-adenylated and capped primary microRNA transcript (pri-miRNA) [12] that can fold into an imperfect stem-loop structure. This structure is recognized by the Dicer-like RNase III endonuclease 1 (DCL1), assisted by the dicing complex core components HYPONASTIC LEAVES 1 (HYL1) and SER-RATE (SE) which promote its cleavage by DCL1, thereby forming a shorter miRNA precursor or pre-miRNA [13,14,15,16]. Plant pre-miRNAs vary in size but rarely exceed 300 nt in length. A pre-miRNA stem contains the mature miRNA strand on one arm and a complementary strand or miRNA-star (miRNA*) on the other. Complementarity between a miRNA and its miRNA* is almost never perfect and may contain up to five mismatched nucleotides, three of which, at most, may form asymmetric bulges [17]. Most pre-miRNAs are further processed by DCL1 into the miRNA/miRNA* duplex [14,15]. This processing is thought to be more precise than that of most siRNA precursors, and this characteristic is used by current *MIR* gene annotation programs to distinguish miRNAs from siRNAs [17].

From the miRNA/miRNA* duplex, usually, the miRNA or guide strand is selected and loaded into the Argonaute (AGO) protein to assemble a miRNA-induced silencing complex (miRISC) while the miRNA* or passenger strand is degraded. Because the majority of miRNAs initiate with uridine, they are incorporated into AGO1, which prefers 5′-uracil-containing small RNA cargoes [18]. The assembled miRISC is guided by the bound miRNA to target mRNAs based on the sequence complementarity between them. Plant miRNAs show high complementarity to their empirically verified mRNA targets throughout their length [19,20]. Upon recognition by miRISC, the target mRNA will be cleaved, its translation will be repressed, or both [21,22,23]; miRNA-guided mRNA cleavage occurs at a precise position in the target mRNA that aligns with the middle of the miRNA (usually between the 10–11th nts) [20,23]. In addition to mRNA cleavage and translational repression, certain 22 nt miRNAs can trigger the production of phased secondary siRNAs (phasiRNAs) from their target transcripts [24].

Tomato (*Solanum lycopersicum*) is an important crop as well as a useful model plant for studying fleshy fruit development and ripening [25]. The tomato genome encodes a single homolog of DCL1 (SlDCL1), which is required for the biogenesis of certain miRNAs [26], and two homologs of Arabidopsis AGO1 (SlAGO1a and SlAGO1b), which are required for the suppression of certain miRNA-targeted mRNAs [27]. Deep sequencing of tomato small RNAs has revealed a complex population of small RNAs, including several conserved miRNAs [26,28,29,30,31,32,33,34,35,36]. Degradome analyses of various tomato tissues have indicated that numerous transcription factors and other regulatory genes are subjected to miRNA-guided cleavage and possibly posttranscriptional regulation [37,38,39,40].

The primary goal of this review is to summarize and curate the results of recent efforts by others and we to annotate the majority of tomato *MIR* genes using the high volume public small RNA sequence data that have accumulated over the years. The resulting non-redundant and exhaustive list of 538 putative and validated *MIR* genes represents a comprehensive tomato *MIR* gene atlas. The atlas contains 169 validated *MIR* genes which code for conserved miRNAs, the properties of which are described in this review. In addition, we critically discuss the functions of tomato miRNAs as inferred from previous gain- and loss-of-function studies. In summary, this review presents a state-of-the-art overview of tomato miRNAs and lays the groundwork for uncovering the functions of uncharacterized miRNAs in this important crop.

## 2. The Canonical miRNA-Coding Loci in the Tomato Genome

The initial studies describing the identification of tomato miRNAs were published in 2007 and 2008 [28,35,41,42,43], before the first draft of the tomato genome was published [44]. In light of this, and the limited small RNA data available at that time, these studies were able to identify conserved miRNAs but had difficulty identifying non-conserved miRNAs. Since the release of the tomato genome in 2012, several efforts have been made to sequence tomato small RNAs under normal and stress conditions and to identify the miRNAs among them [26,29,30,45,46,47,48,49,50,51,52,53,54,55,56]. Despite these efforts, until recently, only 112 tomato *MIR* genes, of which only 61 are defined as non-ambiguous, are found in the latest release (22.1) of the miRBase database [57] (https://www.mirbase.org, accessed on 30 July 2022). Over the years, however, the Sequence Read Archive (SRA; https://www.ncbi.nlm.nih.gov/sra, accessed on 30 July 2022) has accumulated a high volume of sequence data of tomato small RNAs which were extracted from a variety of healthy and infected tissues, including shoots, roots, flowers, and developing and ripening fruits. The SRA data is expected to represent the majority of the tomato miRNome. Recently, three studies and we took advantage of the high volume of SRA deposited small RNA data and the available advanced tomato genome draft sequence (SL3.0) to annotate the full complement of tomato *MIR* genes [58,59,60]. Despite the partial similarity in small RNA data, each study used different small RNA size classes from it as input for different *MIR* annotation algorithms, making them independent studies. Lunardon et al., 2020, used 15–35 nt long small RNAs from 104 data sets as input for the small RNA annotation program ShortStack (designated ShortStack-15-35) [29]; Guo et al., 2020, used 19–24 nt long small RNAs from 30 data sets as input for the *MIR* annotation program miRDeep-P2 [61]; Chen et al., 2021, used 20–22 nt long small RNAs from 91 data sets as input for a custom *MIR* annotation bioinformatic pipeline (designated sRNAanno); like Lunardon et al., 2020, we used the ShortStack program, but unlike him, we used 20–22 nt long small RNAs from 179 tomato small RNAs data sets as input and increased the default pre-miRNA length from 300 nt to 1000 nt (designated ShortStack-20-22). The numbers of unique and shared small RNA datasets used in the different studies are shown in Figure S1A, and data set descriptions are detailed in Table S1. ShortStack-15-35, miRDeep-P2, and sRNAanno annotated 94, 316 (301 following curation), and 260 (259 following curation) candidate miRNA coding loci, respectively. The ShortStack-20-22 analysis resulted in the annotations of 304 putative *MIR* genes. The numbers of shared and unique *MIR* genes annotated in each study are shown in Figure S1B. We compared the resulting *MIR* annotations to previously validated *MIR* genes. It was clear that no study could annotate all *MIR* genes as exemplified for miR156 and miR171 coding genes (Figure S1C). As a result, all annotations from all analyses were compiled into a non-redundant list containing 537 candidate and validated tomato *MIR* genes that code for 415 unique miRNAs (Table S2; hereafter designated as the tomato *MIR* atlas). The aforementioned analyses failed to annotate the previously described sly-miR6023 [5], sly-miR1916, and sly-miR1917 [28]. Sly-miR6023 was included in the atlas but miR1916 and miR1917 were not because of their mixed identity (discussed in Section 3.21 in detail). The folding of all the pre-miRNAs in the atlas is shown in Figure S2.

The miRNAs in the tomato *MIR* atlas can be grouped according to their degree of conservation to conserved (exist in *Solanaceae* as well as in non-*Solanaceae* species; 170 miRNAs; Table S2 marked in purple), lineage-specific (exist only in *Solanaceae* plants; 18 miRNAs; Table S2 marked in orange), and tomato-specific (currently not described in any other plant species; 351 miRNAs; Table S2 marked in red). Since many validated miRNAs are evolutionarily conserved [8], we consider the annotations of *MIR* genes that code for conserved miRNAs as confident. The precision of pre-miRNA processing is expressed as the ratio between the number of small RNAs that correspond to the miRNA and miRNA* and the total number of small RNAs distributed along the pre-miRNA [17]. Interestingly, our “imprecise” ShortStack-20-22 analysis overlapped 97.8% (92/94) of the “precise” ShortStack-15-35 annotations [58] and annotated 61 additional conserved *MIR* genes, 17 of which code for miRNAs that were previously found to require *SlDCL1* for their biogenesis [26] (Table S2), together suggesting that in tomato, the processing of certain miRNA precursors by DCL1 may be less precise than previously thought.

The tomato *MIR* atlas contains 351 *MIR* genes that code for 308 unique tomato-specific miRNAs. As of now, there is no evidence for their conservation in other species, hence we consider the annotations of their *MIR* genes as not confident, especially if annotated by only a single study. Specifically, this applies to 23 *MIR* genes with predicted pre-miRNA folding that do not meet the plant miRNA annotation criteria (Table S2; see Remarks and Figure S2) [17]. On the contrary, several tomato-specific miRNAs exhibit one or more characteristics that support their identity as miRNAs (Figure S1D). For example, 19 were downregulated upon *SlDCL1* silencing (Table S2) [26], suggesting that their precursors are processed by the predominant pre-miRNA processing enzyme. Like most plant miRNAs [8], 205 tomato-specific putative miRNAs have uracil as their first nucleotide (Table S2), supporting their function via AGO1, the major miRNA effector protein [18]. In addition, we found that 301 tomato-specific putative miRNAs have at least one highly complementary tomato cDNA, which might serve as their target mRNA (Table S3). Nevertheless, the identity of the tomato-specific miRNAs must be empirically validated.

### The Conserved miRNA Families in Tomato

**Figure 1:**
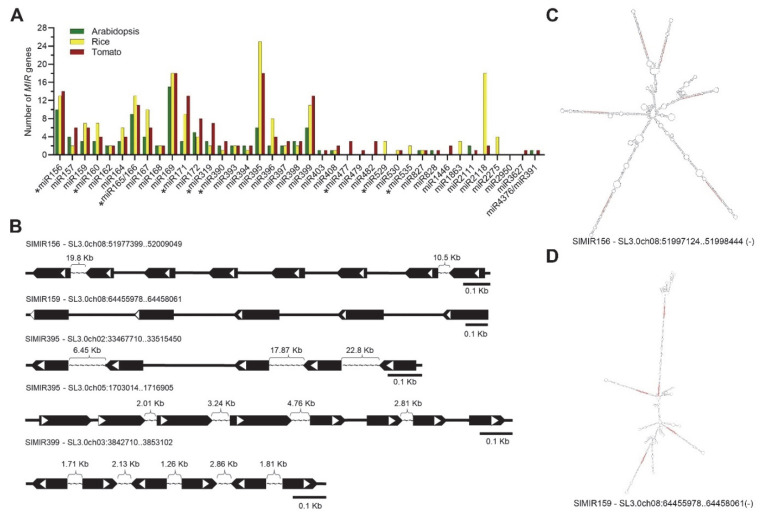
The conserved miRNAs in the tomato genome. (**A**) The number of *MIR* genes that code the indicated conserved miRNAs in tomato, Arabidopsis, and rice (information is based on miRBase release 22 annotations). Deeply conserved miRNAs are marked by *. (**B**) Schematic illustrations of identified *MIR* gene clusters and their locations in the tomato genome. Black pentagons represent predicted pre-miRNA regions and white triangles indicate the locations of respective mature miRNAs. Tilde symbols indicate omitted nucleotides and the distance between two corresponding pre-miRNAs is indicated above them. (**C**,**D**) RNA secondary structures of sly-miR156 (**C**) and sly-miR159 (**D**) polycistronic precursors. The structures were predicted by RNAfold (http://rna.tbi.univie.ac.at/cgi-bin/RNAWebSuite/RNAfold.cgi, accessed on 30 July 2022). The mature miRNA sequence in each hairpin is marked in red. The location of each precursor in the tomato genome (minus indicates reverse complimentary strand) is indicated below.

Recently, 39 plant miRNA families were defined as conserved based on the presence of respective members in at least two major taxonomic divisions [17]. Nine of these families (miR156, miR160, miR166, miR171, miR319, miR390, miR477, miR529, and miR535) have been found to be conserved in most or all of the land plants’ lineages [9,17] and hence dubbed as deeply conserved. Current small RNA data indicate that the tomato genome codes for all of the above-mentioned families, excluding the deeply conserved miR529 and miR535 and the conserved miR1863, miR2275, and miR2950 families (Figure 1A). The conserved and deeply conserved miRNAs are coded in tomatoes by a total of 114 and 54 *MIR* genes, respectively (Table S2). The largest miRNA families in tomatoes are miR395 and miR169, with 18 members in each. These families are also the largest in rice, with 25 and 18 members for miR395 and miR169, respectively. In Arabidopsis, miR169 is the largest family containing 15 members. Compared to Arabidopsis and rice, the tomato miR171, miR172, miR319, and miR399 families are larger and contain 13, 8, 7, and 13 members, respectively (Figure 1A). The updated criteria for plant miRNA annotation limits the pre-miRNA length to 300 nt [17]. Consistent with that, only 2.3% (7/304) of ShortStack-predicted *MIR* genes may be transcribed to significantly longer (≥340 nt) pre-miRNAs. Among them, 3 code for the conserved miRNAs: sly-miR393 (367 nt; Table S2 #235), sly-miR164d (645 nt; Table S2 #441) [62], and sly-miR166 (439 nt; Table S2 #446) (Figure S2). If the long miRNA precursors represent a transient state in *MIR* evolution or an optimal state that has an advantage has yet to be determined.

Four miRNA families, all of which belong to the conserved category, are coded by *MIR* gene clusters (an uninterrupted sequence of 3 or more genes) (Figure 1B). These are miR156, miR159, miR395, and miR399, which are coded by 14, 6, 18, and 13 *MIR* genes, respectively (Table S2). In chromosome 8, miR156 is coded by 9 *MIR* genes, of which, 8 form a ~31.6 Kb cluster that contains a ~1.3 Kb sub-cluster of 6 genes, and miR159 is coded by 5 *MIR* genes that form a ~2.1 Kb cluster (Figure 1B). Moreover, the RNAfold of the sequences of the miR156 sub-cluster and miR159 cluster suggest that they are transcribed as a single polycistronic pri-miRNA (Figure 1C,D). Out of the 18 miR395 coding *MIR* genes, 13 are located within clusters. A total of 5 and 8 *MIR* genes form ~47 Kb and ~13.9 Kb clusters on chromosomes 2 and 5, respectively. On chromosome 3, miR399 is coded by 11 genes, of which, 6 form a ~10 Kb cluster (Figure 1B). It remains to be determined what roles *MIR* gene clustering, and, in particular, pre-miRNA polycistronic co-transcription play in miRNA function.

## 3. The Functions of Tomato miRNAs

Both gain- and loss-of-function approaches have been taken to investigate the functions of tomato miRNAs. To achieve the gain-of-function of a miRNA or its target mRNA, the corresponding pre-miRNA or the cleavage-resistant version of the target mRNA, respectively, were expressed ectopically, mostly under the *CaMV 35S* promoter. When pre-miRNAs are expressed ectopically, their mature miRNAs are overexpressed, which in turn might silence one or more complementary mRNAs. Therefore, this approach may be useful for identifying and functionally analyzing genes that are silenced by miRNAs, but less useful for identifying miRNA functions. The ectopic expression of a cleavage-resistant version of a target mRNA may uncover certain processes that genuinely require its posttranscriptional regulation. However, it may fail to inform all the miRNA-regulated processes, especially if several mRNAs, with either different or redundant functions, are co-targeted by this miRNA. In the case of ectopic transgene expression, both mentioned gain-of-function approaches may be prone to artifacts due to the unrepresentative concentration and expression domains of the transgene that may not fully overlap spatially and temporally with those of the native miRNA or target mRNA. Therefore, when making conclusions about miRNA functions from ectopic expression experiments, caution should be exercised.

Similar to the case of a protein-coding gene, to fully understand the biological function of a miRNA, a loss-of-function approach is considered a prerequisite. In tomatoes, two miRNA loss-of-function approaches have been reported. These involved disrupting the miRNA coding *MIR* gene by CRISPR/Cas9 [63] or knocking down the mature miRNA levels by expressing a target mimic transcript, which sequesters the mature miRNA and contains either a single target mimic site (MIM) [64], two short tandem target mimics (STTM) [65], or multiple target mimics (miRNA sponge). Knockout of a *MIR* gene is the optimal way to decipher the function of its cognate miRNA, especially if the miRNA is coded by a single gene or has a specific function. However, if the studied miRNA belongs to a multi-membered family with functional redundancy, the knockout of a single *MIR* gene may fail to uncover the miRNA function due to functional redundancy. Target mimic has proven useful in examining the function of a multi-membered miRNA family with functional redundancy. However, unlike *MIR* gene knockout, target-mimic-mediated knockdown of a miRNA may fail to uncover the full repertoire of its functions. This is because target mimic activity is relative and depends on the target mimic concentration, its complementarity with the miRNA, and the degree of overlap between its expression domain and the expression domain of the native miRNA [66].

**Figure 2:**
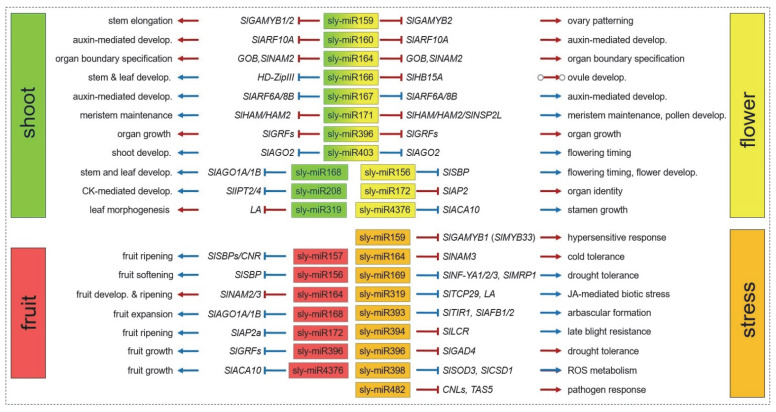
Tomato miRNAs, which were functionally analyzed, their target mRNAs, and the processes they regulate based on the reviewed publications. MiRNAs are color-coded based on their tissue or stress relevance. Red lines and arrows indicate biologically relevant targets and established functions. Blue lines and arrows indicate putative targets and suggested functions. Develop. indicates development.

So far, only 20 tomato miRNAs have been studied by the above-mentioned experimental approaches (Figure 2). In the following sub-sections, we critically review relevant studies and highlight the functions and targets of respective miRNAs that can be inferred from them.

### 3.1. Sly-miR156

The deeply conserved miR156 family is coded by fourteen *MIR* genes in tomatoes, six of which (Table S2 #281, #311, #348, #353, #354, and #356) code for mature miR156, which is identical to Arabidopsis miR156b, and each of the others code for a unique miR156 species (Table S2). Degradome analysis of developing and ripening fruit indicated that seven SQUAMOSA promoter-binding protein (SBP) encoding transcripts, *Colorless non ripening* (*CNR*; *Solyc02g077920*); *SlSBP2* (*Solyc04g045560*); *SlSBP6b* (*Solyc05g012040*); *SlSBP10* (*Solyc05g015510*); *SlSBP13* (*Solyc05g015840*); *Solyc07g062980;* and *SlSBP15* (*Solyc10g078700*), undergo miR156-guided cleavage in the fruit [37]. Consistent with that, ectopic expression of the Arabidopsis gene *MIR156b* in Tomato cv. Micro-Tom resulted in reduced levels of all, except *Solyc07g062980*, and, in addition, downregulated *SlSBP6a* (*Solyc03g114850*), *SlSBP3* (*Solyc10g009080*), and *SlSBP6c* (*Solyc12g038520*) [67,68]. Expression of sly-miR156d (Table S2 #348) in tomato fruits using a PVX virus-vector induced early fruit softening. However, the association of this phenotype with changes in the levels of sly-miR156 and its target mRNAs was not reported [69]. These studies suggest that certain SlSBP-encoding transcripts may be targeted by sly-miR156. However, which transcripts are actually regulated by it, the biological significance of this regulation, and the *MIR* genes involved remain unclear.

### 3.2. Sly-miR157

Related to miR156 in sequence is miR157. In tomato, the miR157 family is coded by six *MIR* genes (Table S2), four of which (Table S2 #83, #104, #162, and #270) code for mature miR157, which is identical to Arabidopsis miR157a, and each of the others code for a unique miR157 species. Two studies have characterized sly-miR157a in tomato using a gain-of-function approach. The transgenic expression of *SlMIR157a* (Table S2 #270) in Tomato cv. Micro-Tom resulted in enhanced sly-miR157a levels and the downregulation of *CNR*, *SlSBP2*, *SlSBP6a*, *SlSBP6b*, *SlSBP3*, and *SlSBP15* in various tissues including young immature fruits, suggesting that their respective transcripts may be subjected to sly-miR157a-guided cleavage. Notably, in that study, *CNR* knockdown in fruits was not associated with their delayed ripening [70]. Meanwhile, the ectopic expression of *SlMIR157a* in young fruits via a virus vector injection led to delayed ripening sectors in 10–20% of the fruits [69]. Consistent with the function of CNR as a ripening promoter [71], these sectors contained increased sly-miR157a precursor and decreased *CNR* levels [69]. The above studies suggest that *CNR* can be targeted by sly-miR157a in the fruit and other tissues. However, as of yet, no mutant of sly-miR157 has been described, and so its involvement in tomato fruit ripening remains an open question.

### 3.3. Sly-miR159

The miR159 family is coded by six *MIR* genes in tomato (Table S2), four of which code for sly-miR159b identical species (Table S2 #378, #379, #380, and #381), one that codes for sly-miR159a (MI0009974; Table S2 #163), and one that codes for sly-miR159 which is identical to ath-miR159a (Table S2 #382). Degradome analysis of developing and ripening fruit pericarp indicated that three SlMYB-encoding transcripts: *SlGAMYB1* (*SlMYB33*, *Solyc01g009070*), *Solyc01g090530*, and *SlGAMYB2* (*Solyc06g073640*) undergo sly-miR159-guided cleavage in the fruit [37]. In addition, a sly-miR159-guided cleavage of *Solyc12g014120*, which encodes a nuclear-localized NOZZLE-domain containing protein with an unknown function, has been demonstrated [72]. The roles of sly-miR159 have been investigated by both gain- and loss-of-function approaches. Ectopic expression of *SlMIR159a* (Table S2 #163) in Tomato cv. Micro-Tom resulted in reduced expression levels of *SlGAMYB1*, *SlGAMYB2*, and *Solyc12g014120* supporting their targeting by sly-miR159 [73]. In another study, tomato plants that ectopically expressed a sly-miR159-resistant version of *Solyc12g014120* exhibited defects in leaf and flower development, including larger multi-locule ovaries, suggesting that sly-miR159-mediated regulation of this target mRNA may be required for normal development [72]. Sly-miR159 silencing in Micro-Tom using a single (35S::MIM159) or tandem target mimic (STTM159) reduced sly-miR159 and increased *SlGAMYB1* and *SlGAMYB2* levels, thus confirming their identity as authentic targets of sly-miR159 [73,74,75]. The vegetative and reproductive development of *35S::MIM159* plants was normal except that they were taller than the control [73]. A similar phenotype was also observed in *STTM159* plants [74], indicating that sly-miR159 may regulate stem elongation through the negative regulation of *SlGAMYB1* and *SlGAMYB2*. In addition, the *STTM159* plants exhibited slightly larger petals and stamens and wider enlarged ovaries with supernumerary carpels that developed into larger wider fruits with more locules. In this study, a similar ovary and fruit phenotype was also observed when a sly-miR159a-resistant version of *SlGAMYB2* was ectopically expressed and in the CRISPR mutant plants that contained 2–5 bp deletions in the *SlMIR159a* precursor backbone. However, the effect of these mutations on the levels of sly-miR159a was not reported [74]. Nonetheless, taken as a whole, these studies indicate that sly-miR159 plays a role in ovary patterning by negatively regulating *SlGAMYB2*. In addition, a recent study demonstrated that the sly-miR159/*SlMYB33* module is involved in geminivirus resistance by regulating the tomato leaf curl New Delhi virus resistance gene *SlSw5a* [75].

### 3.4. Sly-miR160

The tomato genome encompasses four *SlMIR160* genes expressing three mature sly-miR160 species, of which, sly-miR160a, which is coded by two genes (Table S2 #72 and #243) and identical to Arabidopsis miR160a, is the most abundant [76]. Five tomato *Auxin Response Factor* (*ARF*) genes related to the clade of ARF10/ARF16/ARF17 contain a legitimate miR160 targeting site but only *SlARF10A* (*Solyc11g069500*), *SlARF10B* (*Solyc06g073640*), and *SlARF17* (*Solyc11g013470*) have been demonstrated to undergo miR160-guided cleavage [28,37,77]. In line with that, *SlARF10A* expression was upregulated along with *SlARF10B* and *SlARF17* to a lesser extent upon knockdown of miR160 by STTM (STTM160) [76] or through the CRISPR-mediated knockout of *SlMIR160a* (*slmir160a^CR^*) [78], further confirming that they are biologically relevant targets of sly-miR160a. In *STTM160* and *slmir160a^CR^* mutant plants, as well as in transgenic plants ectopically expressing a sly-miR160-resistant *SlARF10A* (*35S::mSlARF10A*), disrupting sly-miR160 regulation similarly affected developmental processes mediated by auxin [79,80,81], such as leaf and floral organ initiation and outgrowth [76,77,78]. A positive correlation has been found between the phenotypic severity of *slmir160a^CR^* mutants and *SlARF10A* expression levels. Consistent with that, the introgression of the *slarf10a^CR^* loss-of-function allele in *slmir160a^CR^* mutants restores leaf and floral organ development [78]. These findings indicate that the fine-tuning of *SlARF10A* by sly-miR160a is critical for auxin-mediated tomato development.

### 3.5. Sly-miR164

There are four *SlMIR164* genes in the tomato genome which code for three mature sly-miR164 species, including one that is identical to Arabidopsis miR164a and is coded by *SlMIR164a* and *SlMIR164b* genes (Table S2 #52 and #397). Four mRNAs encoding NAM/ATAF/CUC-(NAC) domain transcription factors have been demonstrated to undergo miR164-guided cleavage [37,82]. These are the CUC2-like GOBLET (GOB) and SlNAM2, which are involved in organ boundary formation [82,83], SlNAC1, a homolog of NAC1, and SlNAM3, which is most similar to ORESARA1 (ORE1) [84]. Based on their preferential expression in developing shoots and fruits, and their specific phenotypes in CRISPR-mutants, it was concluded that sly-miR164a and sly-miR164b play specialized roles in development. Sly-miR164b knockdown (*slmir164b^CR^*) caused shoot and flower abnormalities, especially supernumerary organs [62,85], reminiscent of plants over-accumulating the boundary genes *GOB* (*Gob4d*) [83] and *SlNAM2* (*mSlNAM2*) [82]. Accordingly, the *slmir164b^CR^* mutant phenotypes were associated with the upregulation of *GOB* and *SlNAM2*, indicating a role for *SlMIR164b* in shoot and flower boundary specification via the negative regulation of corresponding target genes. It has been found that sly-miR164a is preferentially expressed in the fruit pericarp, particularly at the onset and during ripening [62,82]. In Tomato cv. Micro-Tom, evidence that supports sly-miR164a induction by ethylene was provided [85]. However, in tomato cv. Ailsa Craig, sly-miR164a was found to be downregulated in fruits following ethylene treatment [33]. Knockout of *SlMIR164a* caused the upregulation of *SlNAM3*, and to a lesser extent *SlNAM2*, in the ripening fruit pericarp of both tomato cv. M82 and cv. Micro-Tom [62,85], indicating that they serve as sly-miR164a primary targets during ripening. Notably, depletion of sly-miR164a from M82 resulted in smaller fruits with abnormal epidermis at ripening but did not alter the ripening schedule [62], whereas in Micro-Tom, it accelerated ripening [85]. It is concluded that sly-miR164a is required for normal fruit development but that additional evidence is needed to support its involvement in ripening per se. Recently, it was found that sly-miR164a/b silencing via STTM and *SlNAM3* ectopic expression improved cold tolerance in corresponding transgenic plants by promoting ethylene production, suggesting the involvement of the sly-miR164/*SlNAM3* module in tomato cold tolerance [86].

### 3.6. Sly-miR166

Seven *SlMIR166* genes have been annotated in the tomato genome (Table S2), which code for two mature sly-miR166 species, including one that is identical to Arabidopsis lyrata miR166h and is coded by six genes (Table S2 #249, #260, #338, #385, #415, and #424). Degradome analyses indicated that the class III homeodomain-leucine zipper (HD-ZipIII) transcription factors encoding transcripts *Solyc08g066500*, *Solyc12g044410*, and *SlHB15A* (*Solyc03g120910*) undergo miR166-guided cleavage in developing and ripening fruits and open flowers, respectively [37,38], suggesting that they may be regulated by sly-miR166. Consistent with the latter, it has been found that a natural miR166-resistant version of *SlHB15A* (*pf1-6*) accumulates 1.5 fold more *SlHB15A* under cold stress, leading to the development of abnormal ovules and parthenocarpic fruits. This finding suggests that sly-miR166 acts as a cold-inducible switch that regulates *SlHB15A* levels in the ovule [87]. In another study, knockdown of miR166 via STTM led to shorter tomato cv. Micro-Tom plants with abnormally arranged curled leaves suggesting that sly-miR166 plays a role in shoot development [66]. Nevertheless, an analysis of *SlMIR166* gene knockouts is needed to uncover the complete range of sly-miR166 functions.

### 3.7. Sly-miR167

There are seven *SlMIR167* genes in the tomato genome (Table S2) which code for three mature sly-miR167 species, including one that is identical to Arabidopsis miR167a (ath-miR167a) and is coded by four genes (Table S2 #280, #408, #409, and #414). The transgenic expression of *ath-miR167a* in wild tomato (*Solanum pimpinellifolium*) caused increased miR167 accumulation, which was associated with a significant reduction in *SpARF6A* and *SpARF8B* levels, suggesting that they and their tomato homologs may serve as targets of sly-miR167a. The transgenic plants displayed reduced leaf size, shorter internodes, and shorter petals, stamens, and styles, as well as female sterility, suggesting the involvement of SpARF6A and SpARF8B in auxin-mediated lateral-organ growth [88]. Nevertheless, the role of miR167 in tomatoes remains elusive at this time.

### 3.8. Sly-miR168

The tomato genome contains two *SlMIR168* genes, both of which code for identical sly-miR168 species (Table S2 #334 and #532). RNA ligase–mediated-RACE (RLM-RACE) and degradome analyses revealed that *SlAGO1A* (*SlAGO1-1*; *Solyc06g072300*) and *SlAGO1B* (*SlAGO1-2*; *Solyc03g098280*) undergo miR168-guided cleavage in the flower and fruit, respectively, [27,37,89]. In line with that, the apparent knockdown of sly-miR168 in Tomato cv. Micro-Tom by ectopic expression of a miR168-sponge (miR168-SP) resulted in the accumulation of *SlAGO1A* and *SlAGO1B*, further supporting their regulation by sly-miR168 [90]. The miR168-SP plants were slightly shorter but any other developmental abnormalities have not been reported. Consistent with this, the ectopic expression of miR168-resistant *SlAGO1A* (*4m-SlAGO1A*) and *SlAGO1B* (*4m-SlAGO1B*) in Micro-Tom resulted in shorter plants, together indicating that the regulation of *SlAGO1* by sly-miR168 is required for stem elongation. In addition, in that study, the *4m-SlAGO1s* plants were reported to exhibit leaf epinasty and defects in fruit expansion, raising the possibility that the negative posttranscriptional regulation of *SlAGO1s* by sly-miR168 is involved in these processes as well [90], however, this awaits for additional experimental validation.

### 3.9. Sly-miR169

The tomato genome contains 18 *SlMIR169* genes that code for seven distinct sly-miR169 species, of which, the sly-miR169b-like species is coded by eight genes (Table S2). Degradome analysis revealed that four transcripts encoding the Nuclear transcription factor Y (NF-Y) family members: Solyc01g006930, Solyc01g087240, SlNF-YA1 (Solyc08g062210), and Solyc03g121940, undergo miR169-guided cleavage in the fruit [37]. By ectopically expressing the sly-miR169c precursor (Table S2 #299), sly-miR169c accumulation was observed, leading to the downregulation of *SlNF-YA1*, as well as *SlNF-YA2* (*Solyc11g065700*), SlNF-YA3 (*Solyc01g068490*), and the ABC transporter *SlMRP1* (*Solyc09g075020*), suggesting that they serve as targets for sly-miR169 [91]. The sly-miR169c overexpressing plants demonstrated enhanced drought tolerance, indicating the involvement of SlNF-YAs and SlMRP1 [91]. In spite of this, the roles of sly-miR169 are still unknown, and more functional studies are required to uncover them.

### 3.10. Sly-miR171

The tomato genome contains thirteen *SlMIR171* genes that code for nine distinct sly-miR171 species (Table S2). These can be divided into two groups, which are offset by three nucleotides relative to each other, similar to Arabidopsis miR171a and miR171c [92]. RLM-RACE and degradome analyses revealed that the GRAS domain transcription factors encoding transcripts: *SlHAM* (*Solyc08g078800*), *SlHAM2* (*Solyc01g090950*), and *SlNSP2L* (*Solyc11g013150*) undergo miR171-guided cleavage in the flower and fruit [37,40,93]. In line with that, the ectopic expression following transactivation of sly-miR171a precursor (Table S2 #95; *35S>>MIR171a*) and sly-miR171b precursor (Table S2 #89; *35S>>MIR171b*) resulted in higher levels of sly-miR171 and reduced levels of *SlHAM* and *SlHAM2* transcripts. In addition, in *35S>>MIR171b* plants, *SlNSP2L* silencing was observed in accordance with its specific cleavage by sly-miR171b. *SlHAMs* silencing led to meristematic cell overproliferation in meristems and leaf margins, suggesting that they play role in meristem maintenance [93]. Knockdown of sly-miR171a and sly-miR171b by the ectopic expression of STTM171 transcript (*35S::STTM171*) significantly reduced sly-miR171a/b levels and consequently increased the levels of *SlHAM*, *SlHAM2*, and *SlNSP2L* transcripts [92], together confirming that their expression is negatively regulated by sly-miR171. The *35S::STTM171* plants developed irregular compound leaves and excess axillary branches and were male sterile due to abnormal tapetum development that caused the production of malformed and nonviable pollen [92]. These observations suggest that sly-miR171 is involved in tapetum development as well as in shoot development. Which of the sly-miR171 members play roles in these processes and which target gene is regulated by them in each case is yet to be determined.

### 3.11. Sly-miR172

There are eight *SlMIR172* genes in the tomato genome (Table S2) which code for five mature sly-miR172 species, including one that is identical to Arabidopsis miR172a and miR172b and is coded by *SlMIR172a*/*b*/*e*/g (Table S2 #242, #274, #457, and #484). Degradome analyses indicated that the euphyllophyte APETALA2 transcription factors encoding transcripts *SlAP2b* (*Solyc02g064960*), *SlAP2c* (*Solyc02g093150*), *SlAP2a* (*Solyc03g044300*), *Solyc04g049800*, *SlAP2e* (*Solyc06g075510*), *Solyc09g007260*, *Solyc10g084340*, and *SlAP2d* (*Solyc11g072600*) undergo miR172-guided cleavage in flowers and developing and ripening fruit pericarp [37,94]. In tomato, sly-miR172c and sly-miR172d seem the most abundant forms in developing flowers, whereas sly-miR172a/b/e/g is more prevalent in developing and ripening fruits [94]. By the CRISPR-mediated mutagenesis of *SlMIR172c* and *SlMIR172d*, it was found that hypomorphic and loss-of-function mutations in *SlMIR172d*, but not in *SlMIR172c*, converted petals and stamens to sepaloids. Furthermore, mutant flowers displayed graded floral organ abnormalities. These observations suggested a dose-dependent regulation of floral organ identity and number by sly-miR172d, likely through the negative regulation of as yet unknown *AP2* target mRNAs [94]. A gain of function study showed that the ectopic expression of the sly-miR172b precursor decreased the expression of *SlAP2a* [95], which has previously been shown to suppress fruit ripening [96,97,98]. This study and the abundance of sly-miR172a/b/e/g in developing and ripening fruits raise the possibility that sly-miR172 is involved in fruit ripening. To confirm this sly-miR172 role, future studies should focus on the functional analysis of corresponding *SlMIR172* genes preferentially by CRISPR/Cas9-mediated mutagenesis.

### 3.12. Sly-miR208

The tomato genome contains a single *SlMIR208* gene (Table S2 #304). It is noteworthy that sly-miR208 shares the same predicted pre-miRNA as the previously identified putative sly-miR9474 (miRBase MI0029115) and that sly-miR9474 was not annotated as a miRNA by the above analyses and therefore is not included in the tomato *MIR* atlas. Based on sequence complementarity, sly-miR208 was predicted to target *SlIPT2* (*Solyc04g007240*) and *SlIPT4* (*Solyc09g064910*) which encode for isopentenyltransferases that catalyze the initial and rate-limiting step of cytokinin biosynthesis [99,100]. Indeed, the ectopic expression of the sly-miR208 precursor (*35S::pre-miR208*) increased the levels of sly-miR208 and reduced the transcript levels of *SlIPT2* and *SlIPT4* in leaves. Moreover, RLM-RACE revealed that *SlIPT2* and *SlIPT4* encoding transcripts undergo sly-miR208-guided cleavage in the leaves of *35S::pre-miR208* plants, further supporting their targeting by this putative miRNA [101]. However, further research is required to confirm that *SlIPT2* and *SlIPT4* are negatively regulated by the endogenous sly-miR208 and to establish the biological significance of this regulation for cytokinin-mediated tomato development.

### 3.13. Sly-miR319

MiR319 is one of the deeply conserved miRNAs in land plants [10]. There are seven *SlMIR319* genes in the tomato genome (Table S2) which code for four mature sly-miR319 species, including one that is identical to Arabidopsis miR319a and is coded by three genes (Table S2 #248, #383, and #522). Degradome analysis revealed that transcripts encoding seven TEOSINTE BRANCHED1/CYCLOIDEA/PROLIFERATING CELL FACTOR (TCP) family transcription factors: LANCEOLATE (LA; Solyc07g062680), Solyc12g014140, Solyc02g077250, Solyc05g012840, Solyc07g053410, SlTCP29 (Solyc08g048370), and Solyc08g048390 undergo miR319-guided cleavage in the fruit [37]. Consistent with *LA* being a target of sly-miR319, RLM-RACE confirmed that it undergoes sly-miR319-guided cleavage in young leaves and that it is downregulated upon leaf-specific expression of the Arabidopsis miR319a precursor (*FIL>>miR319*) [102]. Moreover, plants homozygous for a gain-of-function mutation in *LA* (*La-2*), which renders it partially resistant to sly-miR319-guided cleavage, and transgenic plants specifically expressing the *La-2* coding region (*LA^m^*) in the leaf primordia (*FIL>>LA^m^*) displayed simple leaf development instead of a large compound leaf [102]. In addition, in situ hybridization indicated that *LA* and miR319 have overlapping expression domains in the margins of the leaf and leaflets primordia, together supporting the conclusion that the fine-tuning of *LA* levels by sly-miR319 in cells is required for tomato compound leaf morphogenesis [102]. Which sly-miR319 coding genes play a role in this process is yet to be determined.

Two studies have provided initial evidence that sly-miR319 regulates Jasmonic acid (JA)-mediated biotic stress responses via its TCP targets. It was found that 24 h post root knot nematode (RKN) infection, sly-miR319b (Table S2 #248) levels declined and, accordingly, the levels of its target *LA* increased, suggesting that sly-miR319b is a negative regulator of the response to RKN infection through *LA*. This suggestion was supported by the fact that RKN resistance increased in *FIL>>LA^m^* and decreased in *FIL>>miR319* tomato plants that accumulated high and low levels of *LA* in leaves, respectively. This is probably due to the induction of JA synthesis genes by LA that in turn increased JA levels and RKN resistance [103]. In the 2nd study, sly-miR319c (Table S2 #148) was found to be upregulated, and accordingly, its target *SlTCP29* was downregulated following *Botrytis cinerea* infection, suggesting that sly-miR319c acts to promote this stress response through the negative regulation of *SlTCP29*. Indeed, the ectopic expression of *SlTCP29* in Arabidopsis improved the resistance of transgenic plants to *B. cinerea* [104]. As a result, the loss-of-function sly-miR319 mutants need to be characterized under stress in order to confirm whether or not endogenous sly-miR319 contributes to the above biotic stress responses.

### 3.14. Sly-miR393

The tomato genome contains two *SlMIR393* genes which code for the same sly-miR393 species as Arabidopsis miR393a (Table S2 #115 and #235). Degradome analysis revealed that transcripts encoding three Transport inhibitor response1 (TIR1)-Auxin-related F box (AFB) family members: SlAFB1 (Solyc02g079190), SlAFB2 (Solyc06g008780), and SlTIR1 (Solyc09g074520), undergo miR393-guided cleavage in the fruit [37]. Treatment of Tomato cv. Micro-Tom roots with auxin analogs stimulated mycorrhization, particularly arbuscular formation. The expression of sly-miR393 was downregulated in Micro-Tom roots inoculated with *Rhizophagus irregularis*, suggesting that miR393 is a negative regulator of the arbuscular formation that is promoted by auxin. In support of that, overexpression of the sly-miR393 precursor (Table S2 #115) in roots reduced its *TIR-AFB* target transcripts and inhibited arbuscule development [105]. Nevertheless, final confirmation for the above roles will be provided by studying mycorrhization in sly-miR393 loss-of-function mutants.

### 3.15. Sly-miR394

The tomato genome contains two genes that code for the same sly-miR394 species that are identical to Arabidopsis miR394a (Table S2 #59 and #202). Degradome analysis revealed that a transcript encoding the F-box protein Leaf Curling Responsiveness (SlLCR; Solyc05g015520) undergoes miR394-guided cleavage in the fruit [37]. Consistent with that, ectopic expression of the sly-miR394 precursor (Table S2 #202) caused a decrease in *SlLCR* levels, while silencing of sly-miR394, via the ectopic expression of STTM transcript (*35S::STTM-miR394*) or the long noncoding RNA40787 (*35S::lncRNA40787*), which functions as endogenous target mimic, downregulated sly-miR394 and in turn induced a strong increase in SlLCR levels [106]. Moreover, the increase in *SlLCR* levels was associated with reduced lesion size on detached leaves inoculated with *Phytophthora infestans*, suggesting that sly-miR394 is a negative regulator of tomato resistance to late blight [106]. A thorough investigation of this function involving the use of knockout mutants and whole plant assay is required to corroborate this role.

### 3.16. Sly-miR396

The tomato genome contains four *SlMIR396* genes, of which, two code for sly-miR396a that is identical to Arabidopsis miR396a (Table S2 #2 and #496), and two code for sly-miR396b (Table S2 #291 and #495). Degradome analysis revealed that seven transcripts encoding GROWTH REGULATING FACTOR (GRF) transcription factors: SlGRF1 (Solyc12g096070), SlGRF2 (Solyc08g005430), SlGRF8 (Solyc03g082430), SlGRF3 (Solyc08g075950), SlGRF7 (Solyc08g083230), SlGRF12 (Solyc10g083510), SlGRF4 (Solyc07g041640), and SlGRF5 (Solyc04g077510) undergo miR396-guided cleavage in the fruit [37]. Knockdown of sly-miR396 via the ectopic expression of target mimicry (STTM396) in Tomato cv. Micro-Tom led to the upregulation of *SlGRF1-7*, depending on the tissue tested, further supporting their regulation by sly-miR396 in planta [107,108]. In line with the role of certain GRFs as positive regulators of cell proliferation [109], the *STTM396* plants developed larger floral organs and larger or elongated fruits [66,107,108], indicating the involvement of sly-miR396 in organ growth via the fine-tuning of GRFs. Examining the physiological and molecular responses to water stress in the sly-miR396 downregulated plants (*MIM396*) revealed that they have higher water use efficiency due to reduced transpiration and a decreased photosynthetic rate. This phenotype was associated with enhanced ABA accumulation and activation of JA and GABA pathways. The latter correlated with increased *GAD4* (*Solyc05g054050*) expression, which was found to undergo sly-miR396 guided cleavage in the fruit pericarp [37] and *MIM396* plants [107]. Thus, sly-miR396 may also act as a negative regulator of drought tolerance in tomatoes.

### 3.17. Sly-miR398

The tomato genome contains three *SlMIR398* genes that code for distinct sly-miR398 species (Table S2 #216, #475, and #539). Degradome analysis revealed that a transcript encoding Superoxide dismutase SlSOD3 (Solyc11g066390) undergoes miR398-guided cleavage in the fruit [37]. The ectopic expression of the sly-miR398 precursor (Table S2 #216) increased the levels of the corresponding miRNA and knocked down the expression of the cytosolic copper/zinc superoxide dismutase *SlCSD1* (*Solyc01g067740*), suggesting that it may be targeted by sly-miR398 [110]. Nevertheless, further research is needed to determine the biological significance of this finding.

### 3.18. Sly-miR403

Sly-miR403 is coded by a single gene in tomato (Table S2 #36). Degradome analysis revealed that Argonaute 2 encoding mRNA (*SlAGO2*; *Solyc02g069260*) undergoes sly-miR403-guided cleavage in the fruit [37]. Ectopic expression of sly-miR403 precursor in Tomato cv. Micro-Tom led to an increase in sly-miR403 and a decrease in *SlAGO2* levels. Two sly-miR403 overexpressing plants exhibited defects in shoot development and delayed flowering time. This suggests that *SlAGO2* may be targeted by sly-miR403 [111]. Nevertheless, further research is needed to determine the biological significance of this finding.

### 3.19. Sly-miR482/sly-miR2118

Previously, six 22 nt long miR482 isoforms (sly-miR482a-f) were identified among tomato seedling’s small RNAs [112], suggesting that the tomato miR482 family contains 6 members. However, only sly-miR482b, c, e sequences could align to the latest version of the tomato genome (SL4.0), and consistently, annotation of sly-miR482 genes identified only three distinct isoforms (Table S2 #197, #263, and #264) [113]. The sly-miR482a and sly-miR482d in miRBase belong to the related sly-miR2118 family [5,112,113] and are named sly-miR2118a and sly-miR2118b, respectively (Table S2 #156 and #262). Degradome analysis revealed that transcripts encoding the Coiled-coil-NBS-LRR (CNL)-type proteins of the plant innate immune system: Solyc11g065780, LRR2 (Solyc04g005540), Solyc09g064610, LRR1 (Solyc02g036270), Solyc07g005770, Solyc07g009180, Solyc08g076000, and Solyc04g009070 undergo miR482-guided cleavage in the fruit [37] and Solyc02g036270, Solyc04g009070, Solyc12g016220, and Solyc05g008070 undergo sly-miR482-guided cleavage in *P. infestans* infected leaves [39]. Consistent with its 22 nt length, sly-miR482-guided cleavage of certain targets induced the formation of phased secondary siRNAs [112,113]. In line with the above, the ectopic expression of sly-miR482b precursor and *STTM482* in tomato led to enhanced and decreased levels of sly-miR482b, respectively, and to opposing change in its target levels in leaves. Moreover, the levels of sly-miR482b negatively correlated with the resistance to *P. infestans* infection in transgenic plants [39]. Similar to sly-miR482b, the ectopic overexpression of the sly-miR482c precursor increased the sensitivity of tomato leaves to *P. infestans*. The downregulation of the *CNL* genes *Solyc07g049700* and *Solyc11g006530* was associated with this phenotype in this study [114]. It has been shown that silencing all sly-miR482 isoforms via ectopic *STTM482* expression reduced the accumulation of sly-miR482-triggered LRR phased secondary siRNAs as well as elevated the expression of *LRR1* and *LRR2*; in addition, these expression changes were associated with enhanced resistance to *P. infestans* and *Ps. syringae* pv. tomato DC3000 [113]. In an additional study, the Cas9-mediated mutagenesis of the *SlMIR482e* gene (*slmir482e^CR^*) knocked down sly-miR482e levels and in turn increased the levels of *Soly08g075630* and *Soly08g076000*. Moreover, the *slmir482e^CR^* plants displayed enhanced resistance when inoculated with *Fusarium oxysporum* f. sp. lycopersici (race 2) [115]. Taken together, the above studies suggest that sly-miR482 family members are involved in the response to various pathogen infections by regulating the expression levels of certain CNL-type resistance proteins. With regard to sly-miR2118, silencing sly-miR2118b via ectopic STTM expression was associated with the upregulation of its predicted target *TAS5* and increased resistance to *P. infestans*, suggesting that sly-miR2118b is also involved in the response to pathogens [113].

The tomato miRNA sly-miR-W and its star sequence sly-miR-W* were initially cloned from Tomato cv. Micro-Tom tissues [116]. Annotation of sly-miR-W suggests that it is identical to sly-miR482e* and that sly-miR-W* is identical to sly-miR482e (Table S2 #197). RLM-RACE suggested that sly-miR-W*/sly-miR482 guides the cleavage of two target genes encoding membrane-bound ATPase (*SGN-U573791*) and glutamate permease (*SGN-U585460*), both of which are associated with glutamate accumulation [116]. At present, additional evidence that these putative target genes are regulated by sly-miR482 was not published. Thus, whether sly-miR482 also regulates glutamate transport currently remains an open question.

### 3.20. Sly-miR858

Previously, Jia et al., 2015 identified the tomato homolog of miR858 (sly-miR858) and demonstrated by RT-qPCR that it is ubiquitously expressed, and by RLM-RACE that it guides the cleavage of several mRNAs encoding R2R3 MYB transcription factors. Moreover, the silencing of sly-miR858 using STTM elevated targeted *MYB* transcripts and induced anthocyanin accumulation in tomatoes, together suggesting that sly-miR858 regulates anthocyanin biosynthesis [117]. However, BLAST analysis indicated that the identified sly-miR858 sequence could not align with the latest version of the tomato genome (SL4.0), which explains why it was not annotated as a miRNA. Interestingly, Cháves Montes et al., 2013, detected among tomato small RNAs several *Solanaceae*-specific small RNAs that are highly similar but not identical to the identified sly-miR858 [8]. This may explain the results of Jia et al., 2015, and raises the possibility that they are involved in the regulation of anthocyanin biosynthesis.

### 3.21. Sly-miR1916/sly-miR1917

The small RNAs miR1916 and miR1917 were originally cloned from Tomato c.v. Micro-Tom and were annotated as new tomato miRNAs based on their expression, the presence of complementary mRNAs, which may serve as their target genes, and the formation of hairpin structures from their surrounding genomic sequences. These putative *MIR* genes, both of which are located on chromosome 8, were deposited to miRBase under accession numbers *MI0008351* (*SlMIR1916*) and *MI0008352* (*SlMIR1917*) [28]. The ectopic expression of miR1916 and miR1917 hairpin sequences in tomato increased the levels of corresponding small RNAs, thus supporting their identity as their pre-miRNAs [118,119]. However, to date, miR1916* and miR1917* strands were not cloned, and accordingly, sly-miR1916 and sly-miR1917 were not annotated as miRNA in this review. In fact, sly-miR1916 and sly-miR1917 were annotated by sRNAanno as phased siRNAs that are produced from *PHAS21-110* (SL3.0ch12:67449889-67451108) and *PHAS24-19* (SL3.0ch01: 3661062-3662189) transcripts, respectively [60]. In line with this, the examination of the current tomato genome (SL4.0) with sly-miR1916 and sly-miR1917 sequences revealed 45 and 60 identical loci, respectively; a number that is much higher than that of any other tomato validated miRNA (Figure 1A). Target prediction suggested *SGN-U322371* and *SlCTR4* splice variants of *SlCTR4sv1-3* as targets for sly-miR1916 and sly-miR1917, respectively [28]. In a recent study, sly-miR1916 was suggested to guide the cleavage of *STR-1*, *UGT*, *R1B-16*, and *MYB12* involved in the response against *Phytophthora infestans* and *Botrytis cinerea* [118]. However, in both studies, sly-miR1916 target validation by RLM-RACE did not reveal a canonical cleavage site, which aligns with its 10–11th nucleotides [28,118]. For sly-miR1917, a *LeCTR4sv1* legitimate cleavage product was identified by RLM-RACE [28]. Nevertheless, the degradome analysis of developing and ripening fruit, which were shown to express sly-miR1916 and sly-miR1917 [28], did not reveal significant cleavage products for both [37]. Taken together, the available data regarding sly-miR1916 and sly-miR1917 are not conclusive and further studies are required to determine whether they are miRNAs, siRNAs, or maybe both.

### 3.22. Sly-miR4376

Sly-miR4376 (also known as sly-miR391) is a 22 nt miRNA that is coded by a single gene in the tomato genome (Table S2 #252). RLM-RACE indicated that the autoinhibited Ca^2+^-ATPase (*SlACA10*; *Solyc07g008320*) mRNA is subjected to sly-miR4376-mediated cleavage that in turn induces the formation of secondary phased siRNAs from the cleaved transcript. The ectopic expression of the sly-miR4376 precursor resulted in elevated levels of sly-miR4376 and reduced the accumulation of *SlACA10* mRNA, further supporting its targeting by sly-miR4376. The ectopic expression of the sly-miR4376-resistant version of *SlACA10* (*35S::SlACA10^R^*) caused the accumulation of respective transcripts in *35S::SlACA10^R^* plants. This was associated with *35S::SlACA10^R^* elongated stamen filaments and the drastically reduced yield of apparently normal-looking fruits, likely due to inhibition of young fruit growth. Interestingly, a similar instead of opposite phenotype was also observed in the sly-miR4376 overexpressing plants, together raising the possibility that the fine-tuning of *SlACA10* expression by sly-miR4376 is important for tomato reproductive development [120]. Nevertheless, the characterization of a loss-of-function sly-miR4376 mutant is required to confirm this possibility.

## 4. Conclusions and Perspectives

In the past decade, numerous labs have cloned and sequenced small RNAs from various tomato tissues under diverse physiological conditions, thereby enabling the discovery of the lion’s share of its small RNAs, including miRNAs. Using this large volume of small RNA data, *MIR* genes were annotated [58,59,60], revealing at least 169 validated *MIR* genes coding for conserved miRNAs, 18 putative *MIR* genes coding for *Solanaceae*-specific miRNAs, and 351 putative *MIR* genes coding for miRNAs specific to tomatoes. The tomato genome coded for 34 conserved miRNA families, of which, 7 are deeply conserved. Of them, the miR156, miR159, miR395, and miR399 families are coded by *MIR* gene clusters, and the sly-miR393, sly-miR164, and sly-miR166 families contain members that are coded by unconventionally long miRNA precursors. The significance of clustering and long pre-miRNAs is yet to be discovered. The largest tomato miRNA families are the conserved miR395 and miR169, similar to rice and Arabidopsis in the case of miR169. Despite extensive efforts to identify tomato miRNAs, at present, the functions of most miRNAs remain understudied in this scientifically and biotechnologically important model crop. Fewer than twenty miRNAs have been fully characterized and only a handful of their functions have been linked to a respective *MIR* gene. In particular, there is a lack of solid evidence on the involvement of miRNAs in tomato fleshy fruit ripening. Current data suggest that tomato miRNAs play roles in the shoot, flower, and fruit development as well as in the regulation of biotic and abiotic stress responses. Several established miRNA roles are conserved, such as miR160’s in auxin-mediated development, miR164’s in boundary specification, miR172’s in floral organ identity, and miR396’s in organ growth. However, others are not, such as miR164’s role in fruit ripening and miR159’s role in the hypersensitive response. The specific functions of tomato miRNAs, as well as the numerous uncharacterized miRNAs, clearly indicate that there is still much to be learn about tomato miRNAs. Using the recently introduced CRISPR/Cas9 genome editing technology, which can be easily applied to tomatoes [62,63,78], it is now possible to decipher the specific function of each and every tomato *MIR* gene. Such a genome-wide approach will surely improve our understanding of tomato development and stress and especially how the complex process of fleshy fruit development and ripening is controlled. It is also expected to uncover biotechnologically useful *MIR* alleles and miRNA target genes that can be used to improve tomato and potentially other *Solanaceae* crops.

## Supplementary Materials

The following are available online at https://www.mdpi.com/article/10.3390/ijms231911979/s1.

## Abbreviations

Cas9	CRISPR associated protein9
CNR	Colorless non ripening
CRISPR	Clustered Regularly Interspaced Short Palindromic Repeats
DCL1	Dicer-like RNase III endonuclease 1
HYL1	HYPONASTIC LEAVES 1
JA	Jasmonic acid
MIR	MiRNA-coding gene
miRNA	MicroRNA
miRNA*	MiRNA star or passenger strand
miRISC	MiRNA-induced silencing complex
pre-miRNA	MiRNA precursor
pri-miRNA	Primary microRNA transcript
RLM-RACE	RNA ligase–mediated RACE
SE	SERRATE
SRA	Sequence Read Archive

## References

[1] Borges F., Martienssen R.A. (2015). The Expanding World of Small RNAs in Plants. Nat. Rev. Mol. Cell Biol..

[2] Axtell M.J. (2013). Classification and Comparison of Small RNAs from Plants. Annu. Rev. Plant Biol..

[3] Chen X. (2009). Small RNAs and Their Roles in Plant Development. Annu. Rev. Cell Dev..

[4] Sunkar R., Li Y.-F., Jagadeeswaran G. (2012). Functions of MicroRNAs in Plant Stress Responses. Trends Plant Sci..

[5] Li F., Pignatta D., Bendix C., Brunkard J.O., Cohn M.M., Tung J., Sun H., Kumar P., Baker B. (2012). MicroRNA Regulation of Plant Innate Immune Receptors. Proc. Natl. Acad. Sci. USA.

[6] Song X., Li Y., Cao X., Qi Y. (2019). MicroRNAs and Their Regulatory Roles in Plant–Environment Interactions. Annu. Rev. Plant Biol..

[7] Wu L., Zhou H., Zhang Q., Zhang J., Ni F., Liu C., Qi Y. (2010). DNA Methylation Mediated by a MicroRNA Pathway. Mol. Cell.

[8] Chávez Montes R.A., Rosas-Cárdenas D.F., De Paoli E., Accerbi M., Rymarquis L.A., Mahalingam G., Marsch-Martínez N., Meyers B.C., Green P.J., de Folter S. (2014). Sample Sequencing of Vascular Plants Demonstrates Widespread Conservation and Divergence of MicroRNAs. Nat. Commun..

[9] Axtell M.J., Bowman J.L. (2008). Evolution of Plant MicroRNAs and Their Targets. Trends Plant Sci..

[10] Arazi T., Talmor-Neiman M., Stav R., Riese M., Huijser P., Baulcombe D.C. (2005). Cloning and Characterization of Micro-RNAs from Moss: MiRNAs in Moss. Plant J..

[11] You C., Cui J., Wang H., Qi X., Kuo L.-Y., Ma H., Gao L., Mo B., Chen X. (2017). Conservation and Divergence of Small RNA Pathways and MicroRNAs in Land Plants. Genome Biol..

[12] Xie Z., Allen E., Fahlgren N., Calamar A., Givan S.A., Carrington J.C. (2005). Expression of Arabidopsis MIRNA Genes. Plant Physiol..

[13] Park W., Li J., Song R., Messing J., Chen X. (2002). Carpel factory, a Dicer Homolog, and HEN1, a Novel Protein, Act in MicroRNA Metabolism in Arabidopsis Thaliana. Curr. Biol. CB.

[14] Kurihara Y., Watanabe Y. (2004). Arabidopsis Micro-RNA Biogenesis through Dicer-like 1 Protein Functions. Proc. Natl. Acad. Sci. USA.

[15] Yu Y., Jia T., Chen X. (2017). The ‘How’ and ‘Where’ of Plant MicroRNAs. New Phytol..

[16] Fang Y., Spector D.L. (2007). Identification of Nuclear Dicing Bodies Containing Proteins for MicroRNA Biogenesis in Living Arabidopsis Plants. Curr. Biol..

[17] Axtell M.J., Meyers B.C. (2018). Revisiting Criteria for Plant MiRNA Annotation in the Era of Big Data. Plant Cell.

[18] Mi S., Cai T., Hu Y., Chen Y., Hodges E., Ni F., Wu L., Li S., Zhou H., Long C. (2008). Sorting of Small RNAs into Arabidopsis Argonaute Complexes Is Directed by the 5′ Terminal Nucleotide. Cell.

[19] Jones-Rhoades M.W., Bartel D.P. (2004). Computational Identification of Plant MicroRNAs and Their Targets, Including a Stress-Induced MiRNA. Mol. Cell.

[20] Schwab R., Palatnik J.F., Riester M., Schommer C., Schmid M., Weigel D. (2005). Specific Effects of MicroRNAs on the Plant Transcriptome. Dev. Cell.

[21] Brodersen P., Sakvarelidze-Achard L., Bruun-Rasmussen M., Dunoyer P., Yamamoto Y.Y., Sieburth L., Voinnet O. (2008). Widespread Translational Inhibition by Plant MiRNAs and SiRNAs. Science.

[22] Li S., Liu L., Zhuang X., Yu Y., Liu X., Cui X., Ji L., Pan Z., Cao X., Mo B. (2013). MicroRNAs Inhibit the Translation of Target MRNAs on the Endoplasmic Reticulum in Arabidopsis. Cell.

[23] Llave C., Xie Z., Kasschau K.D., Carrington J.C. (2002). Cleavage of Scarecrow-like MRNA Targets Directed by a Class of Arabidopsis MiRNA. Science.

[24] Chen H.M., Chen L.T., Patel K., Li Y.H., Baulcombe D.C., Wu S.H. (2010). 22-Nucleotide RNAs Trigger Secondary SiRNA Biogenesis in Plants. Proc. Natl. Acad. Sci. USA.

[25] Seymour G.B., Østergaard L., Chapman N.H., Knapp S., Martin C. (2013). Fruit Development and Ripening. Annu. Rev. Plant Biol..

[26] Kravchik M., Sunkar R., Damodharan S., Stav R., Zohar M., Isaacson T., Arazi T. (2014). Global and Local Perturbation of the Tomato MicroRNA Pathway by a Trans-Activated DICER-LIKE 1 Mutant. J. Exp. Bot..

[27] Hendelman A., Kravchik M., Stav R., Zik M., Lugassi N., Arazi T. (2012). The Developmental Outcomes of P0-Mediated ARGONAUTE Destabilization in Tomato. Planta.

[28] Moxon S., Jing R., Szittya G., Schwach F., Rusholme Pilcher R.L., Moulton V., Dalmay T. (2008). Deep Sequencing of Tomato Short RNAs Identifies MicroRNAs Targeting Genes Involved in Fruit Ripening. Genome Res..

[29] Axtell M.J. (2013). ShortStack: Comprehensive Annotation and Quantification of Small RNA Genes. RNA.

[30] Zuo J., Fu D., Zhu Y., Qu G., Tian H., Zhai B., Ju Z., Gao C., Wang Y., Luo Y. (2013). SRNAome Parsing Yields Insights into Tomato Fruit Ripening Control. Physiol. Plant.

[31] Kravchik M., Damodharan S., Stav R., Arazi T. (2014). Generation and Characterization of a Tomato DCL3-Silencing Mutant. Plant Sci..

[32] Jin W., Wu F. (2015). Characterization of MiRNAs Associated with Botrytis Cinerea Infection of Tomato Leaves. BMC Plant Biol..

[33] Gao C., Ju Z., Cao D., Zhai B., Qin G., Zhu H., Fu D., Luo Y., Zhu B. (2015). MicroRNA Profiling Analysis throughout Tomato Fruit Development and Ripening Reveals Potential Regulatory Role of RIN on MicroRNAs Accumulation. Plant Biotechnol. J..

[34] Kaur P., Shukla N., Joshi G., VijayaKumar C., Jagannath A., Agarwal M., Goel S., Kumar A. (2017). Genome-Wide Identification and Characterization of MiRNAome from Tomato (*Solanum Lycopersicum*) Roots and Root-Knot Nematode (*Meloidogyne Incognita*) during Susceptible Interaction. PLoS ONE.

[35] Itaya A., Bundschuh R., Archual A.J., Joung J.-G., Fei Z., Dai X., Zhao P.X., Tang Y., Nelson R.S., Ding B. (2008). Small RNAs in Tomato Fruit and Leaf Development. Biochim. Biophys. Acta-Gene Regul. Mech..

[36] Cardoso T.C.d.S., Alves T.C., Caneschi C.M., Santana D.d.R.G., Fernandes-Brum C.N., Reis G.L.D., Daude M.M., Ribeiro T.H.C., Gómez M.M.D., Lima A.A. (2018). New Insights into Tomato MicroRNAs. Sci. Rep..

[37] Karlova R., van Haarst J.C., Maliepaard C., van de Geest H., Bovy A.G., Lammers M., Angenent G.C., de Maagd R.A. (2013). Identification of MicroRNA Targets in Tomato Fruit Development Using High-Throughput Sequencing and Degradome Analysis. J. Exp. Bot..

[38] Lopez-Gomollon S., Mohorianu I., Szittya G., Moulton V., Dalmay T. (2012). Diverse Correlation Patterns between MicroRNAs and Their Targets during Tomato Fruit Development Indicates Different Modes of MicroRNA Actions. Planta.

[39] Jiang N., Meng J., Cui J., Sun G., Luan Y. (2018). Function Identification of MiR482b, a Negative Regulator during Tomato Resistance to Phytophthora Infestans. Hortic. Res..

[40] Huang W., Xian Z., Kang X., Tang N., Li Z. (2015). Genome-Wide Identification, Phylogeny and Expression Analysis of GRAS Gene Family in Tomato. BMC Plant Biol..

[41] Pilcher R.L.R., Moxon S., Pakseresht N., Moulton V., Manning K., Seymour G., Dalmay T. (2007). Identification of Novel Small RNAs in Tomato (*Solanum Lycopersicum*). Planta.

[42] Zhang J., Zeng R., Chen J., Liu X., Liao Q. (2008). Identification of Conserved MicroRNAs and Their Targets from *Solanum Lycopersicum* Mill. Gene.

[43] Yin Z., Li C., Han X., Shen F. (2008). Identification of Conserved MicroRNAs and Their Target Genes in Tomato (*Lycopersicon Esculentum*). Gene.

[44] Sato S., Tabata S., Hirakawa H., Asamizu E., Shirasawa K., Isobe S., Kaneko T., Nakamura Y., Shibata D., Aoki K. (2012). The Tomato Genome Sequence Provides Insights into Fleshy Fruit Evolution. Nature.

[45] Zuo J., Zhu B., Fu D., Zhu Y., Ma Y., Chi L., Ju Z., Wang Y., Zhai B., Luo Y. (2012). Sculpting the Maturation, Softening and Ethylene Pathway: The Influences of MicroRNAs on Tomato Fruits. BMC Genom..

[46] Li F., Orban R., Baker B. (2012). SoMART: A Web Server for Plant MiRNA, TasiRNA and Target Gene Analysis: Web Tools for MiRNA and TasiRNA Analysis. Plant J..

[47] Xu D., Guo S., Liu M. (2013). Identification of MiRNAs Involved in Long-Term Simulated Microgravity Response in *Solanum lycopersicum*. Plant Physiol. Biochem..

[48] Din M., Barozai M.Y.K. (2014). Profiling MicroRNAs and Their Targets in an Important Fleshy Fruit: Tomato (*Solanum Lycopersicum*). Gene.

[49] Bokszczanin K.L., Krezdorn N., Fragkostefanakis S., Müller S., Rycak L., Chen Y., Hoffmeier K., Kreutz J., Paupière M.J., Chaturvedi P. (2015). Identification of Novel Small NcRNAs in Pollen of Tomato. BMC Genom..

[50] Liu M., Yu H., Zhao G., Huang Q., Lu Y., Ouyang B. (2018). Identification of Drought-Responsive MicroRNAs in Tomato Using High-Throughput Sequencing. Funct. Integr. Genom..

[51] Pan C., Ye L., Zheng Y., Wang Y., Yang D., Liu X., Chen L., Zhang Y., Fei Z., Lu G. (2017). Identification and Expression Profiling of MicroRNAs Involved in the Stigma Exsertion under High-Temperature Stress in Tomato. BMC Genom..

[52] Liu M., Yu H., Zhao G., Huang Q., Lu Y., Ouyang B. (2017). Profiling of Drought-Responsive MicroRNA and MRNA in Tomato Using High-Throughput Sequencing. BMC Genom..

[53] Zuo J., Wang Q., Han C., Ju Z., Cao D., Zhu B., Luo Y., Gao L. (2017). SRNAome and Degradome Sequencing Analysis Reveals Specific Regulation of SRNA in Response to Chilling Injury in Tomato Fruit. Physiol. Plant.

[54] Wang Y., Wang Q., Gao L., Zhu B., Ju Z., Luo Y., Zuo J. (2017). Parsing the Regulatory Network between Small RNAs and Target Genes in Ethylene Pathway in Tomato. Front. Plant Sci..

[55] Candar-Cakir B., Arican E., Zhang B. (2016). Small RNA and Degradome Deep Sequencing Reveals Drought-and Tissue-Specific Micrornas and Their Important Roles in Drought-Sensitive and Drought-Tolerant Tomato Genotypes. Plant Biotechnol. J..

[56] Zhang Y., Wang Y., Xie F., Li C., Zhang B., Nichols R.L., Pan X. (2016). Identification and Characterization of MicroRNAs in the Plant Parasitic Root-Knot Nematode Meloidogyne Incognita Using Deep Sequencing. Funct. Integr. Genom..

[57] Kozomara A., Griffiths-Jones S. (2014). MiRBase: Annotating High Confidence MicroRNAs Using Deep Sequencing Data. Nucleic Acids Res.

[58] Lunardon A., Johnson N.R., Hagerott E., Phifer T., Polydore S., Coruh C., Axtell M.J. (2020). Integrated Annotations and Analyses of Small RNA–Producing Loci from 47 Diverse Plants. Genome Res..

[59] Guo Z., Kuang Z., Wang Y., Zhao Y., Tao Y., Cheng C., Yang J., Lu X., Hao C., Wang T. (2020). PmiREN: A Comprehensive Encyclopedia of Plant MiRNAs. Nucleic Acids Res..

[60] Chen C., Li J., Feng J., Liu B., Feng L., Yu X., Li G., Zhai J., Meyers B.C., Xia R. (2021). SRNAanno—A Database Repository of Uniformly Annotated Small RNAs in Plants. Hortic. Res..

[61] Kuang Z., Wang Y., Li L., Yang X. (2019). MiRDeep-P2: Accurate and Fast Analysis of the MicroRNA Transcriptome in Plants. Bioinformatics.

[62] Gupta S.K., Vishwakarma A., Kenea H.D., Galsurker O., Cohen H., Aharoni A., Arazi T. (2021). CRISPR/Cas9 Mutants of Tomato *MICRORNA164* Genes Uncover Their Functional Specialization in Development. Plant Physiol..

[63] Brooks C., Nekrasov V., Lippman Z., Eck J.V. (2014). Efficient Gene Editing in Tomato in the First Generation Using the CRISPR/Cas9 System. Plant Physiol..

[64] Franco-Zorrilla J.M., Valli A., Todesco M., Mateos I., Puga M.I., Rubio-Somoza I., Leyva A., Weigel D., García J.A., Paz-Ares J. (2007). Target Mimicry Provides a New Mechanism for Regulation of MicroRNA Activity. Nat. Genet..

[65] Yan J., Gu Y., Jia X., Kang W., Pan S., Tang X., Chen X., Tang G. (2012). Effective Small RNA Destruction by the Expression of a Short Tandem Target Mimic in Arabidopsis. Plant Cell.

[66] Peng T., Qiao M., Liu H., Teotia S., Zhang Z., Zhao Y., Wang B., Zhao D., Shi L., Zhang C. (2018). A Resource for Inactivation of MicroRNAs Using Short Tandem Target Mimic Technology in Model and Crop Plants. Mol. Plant.

[67] Silva G.F.F.E., Silva E.M., da Silva Azevedo M., Guivin M.A.C., Ramiro D.A., Figueiredo C.R., Carrer H., Peres L.E.P., Nogueira F.T.S. (2014). MicroRNA156-Targeted SPL/SBP Box Transcription Factors Regulate Tomato Ovary and Fruit Development. Plant J..

[68] Silva G.F.F., Silva E.M., Correa J.P.O., Vicente M.H., Jiang N., Notini M.M., Junior A.C., De Jesus F.A., Castilho P., Carrera E. (2019). Tomato Floral Induction and Flower Development Are Orchestrated by the Interplay between Gibberellin and Two Unrelated MicroRNA-Controlled Modules. New Phytol..

[69] Chen W., Kong J., Lai T., Manning K., Wu C., Wang Y., Qin C., Li B., Yu Z., Zhang X. (2015). Tuning *LeSPL-CNR* Expression by SlymiR157 Affects Tomato Fruit Ripening. Sci. Rep..

[70] Zhang X., Zou Z., Zhang J., Zhang Y., Han Q., Hu T., Xu X., Liu H., Li H., Ye Z. (2010). Over-Expression of Sly-MiR156a in Tomato Results in Multiple Vegetative and Reproductive Trait Alterations and Partial Phenocopy of the Sft Mutant. FEBS Lett..

[71] Manning K., Tör M., Poole M., Hong Y., Thompson A.J., King G.J., Giovannoni J.J., Seymour G.B. (2006). A Naturally Occurring Epigenetic Mutation in a Gene Encoding an SBP-Box Transcription Factor Inhibits Tomato Fruit Ripening. Nat. Genet..

[72] Buxdorf K., Hendelman A., Stav R., Lapidot M., Ori N., Arazi T. (2010). Identification and Characterization of a Novel MiR159 Target Not Related to MYB in Tomato. Planta.

[73] da Silva E.M., Silva G.F.F.E., Bidoia D.B., da Silva Azevedo M., de Jesus F.A., Pino L.E., Peres L.E.P., Carrera E., López-Díaz I., Nogueira F.T.S. (2017). MicroRNA159-Targeted SlGAMYB Transcription Factors Are Required for Fruit Set in Tomato. Plant J..

[74] Zhao P., Wang F., Deng Y., Zhong F., Tian P., Lin D., Deng J., Zhang Y., Huang T. (2022). Sly-MiR159 Regulates Fruit Morphology by Modulating GA Biosynthesis in Tomato. Plant Biotechnol. J..

[75] Sharma N., Sahu P.P., Prasad A., Muthamilarasan M., Waseem M., Khan Y., Thakur J.K., Chakraborty S., Prasad M. (2021). The Sw5a Gene Confers Resistance to ToLCNDV and Triggers an HR Response after Direct AC4 Effector Recognition. Proc. Natl. Acad. Sci. USA.

[76] Damodharan S., Zhao D., Arazi T. (2016). A Common MiRNA160-Based Mechanism Regulates Ovary Patterning, Floral Organ Abscission and Lamina Outgrowth in Tomato. Plant J..

[77] Hendelman A., Buxdorf K., Stav R., Kravchik M., Arazi T. (2012). Inhibition of Lamina Outgrowth Following *Solanum Lycopersicum* auxin response factor 10 (SlARF10) Derepression. Plant Mol. Biol..

[78] Damodharan S., Corem S., Gupta S.K., Arazi T. (2018). Tuning of SlARF10A Dosage by Sly-MiR160a Is Critical for Auxin-Mediated Compound Leaf and Flower Development. Plant J..

[79] Reinhardt D., Mandel T., Kuhlemeier C. (2000). Auxin Regulates the Initiation and Radial Position of Plant Lateral Organs. Plant Cell.

[80] Koenig D., Bayer E., Kang J., Kuhlemeier C., Sinha N. (2009). Auxin Patterns *Solanum Lycopersicum* Leaf Morphogenesis. Development.

[81] Cheng Y., Dai X., Zhao Y. (2006). Auxin Biosynthesis by the YUCCA Flavin Monooxygenases Controls the Formation of Floral Organs and Vascular Tissues in Arabidopsis. Genes Dev..

[82] Hendelman A., Stav R., Zemach H., Arazi T. (2013). The Tomato NAC Transcription Factor SlNAM2 Is Involved in Flower-Boundary Morphogenesis. J. Exp. Bot..

[83] Berger Y., Harpaz-Saad S., Brand A., Melnik H., Sirding N., Alvarez J.P., Zinder M., Samach A., Eshed Y., Ori N. (2009). The NAC-Domain Transcription Factor GOBLET Specifies Leaflet Boundaries in Compound Tomato Leaves. Development.

[84] Kim J.H., Woo H.R., Kim J., Lim P.O., Lee I.C., Choi S.H., Hwang D., Nam H.G. (2009). Trifurcate Feed-Forward Regulation of Age-Dependent Cell Death Involving MiR164 in Arabidopsis. Science.

[85] Lin D., Zhu X., Qi B., Gao Z., Tian P., Li Z., Lin Z., Zhang Y., Huang T. (2022). SlMIR164A Regulates Fruit Ripening and Quality by Controlling SlNAM2 and SlNAM3 in Tomato. Plant Biotechnol. J..

[86] Dong Y., Tang M., Huang Z., Song J., Xu J., Ahammed G.J., Yu J., Zhou Y. (2022). The MiR164a-NAM3 Module Confers Cold Tolerance by Inducing Ethylene Production in Tomato. Plant J..

[87] Clepet C., Devani R.S., Boumlik R., Hao Y., Morin H., Marcel F., Verdenaud M., Mania B., Brisou G., Citerne S. (2021). The MiR166–SlHB15A Regulatory Module Controls Ovule Development and Parthenocarpic Fruit Set under Adverse Temperatures in Tomato. Mol. Plant.

[88] Liu N., Wu S., Van Houten J., Wang Y., Ding B., Fei Z., Clarke T.H., Reed J.W., van der Knaap E. (2014). Down-Regulation of auxin response factors 6 and 8 by MicroRNA 167 Leads to Floral Development Defects and Female Sterility in Tomato. J. Exp. Bot..

[89] Xian Z., Yang Y., Huang W., Tang N., Wang X., Li Z. (2013). Molecular Cloning and Characterisation of SlAGO family in Tomato. BMC Plant Biol..

[90] Xian Z., Huang W., Yang Y., Tang N., Zhang C., Ren M., Li Z. (2014). MiR168 Influences Phase Transition, Leaf Epinasty, and Fruit Development via SlAGO1s in Tomato. J. Exp. Bot..

[91] Zhang X., Zou Z., Gong P., Zhang J., Ziaf K., Li H., Xiao F., Ye Z. (2011). Over-Expression of MicroRNA169 Confers Enhanced Drought Tolerance to Tomato. Biotechnol. Lett..

[92] Kravchik M., Stav R., Belausov E., Arazi T. (2019). Functional Characterization of MicroRNA171 Family in Tomato. Plants.

[93] Hendelman A., Kravchik M., Stav R., Frank W., Arazi T. (2016). Tomato Hairy Meristem Genes Are Involved in Meristem Maintenance and Compound Leaf Morphogenesis. J. Exp. Bot..

[94] Lin W., Gupta S.K., Arazi T., Spitzer-Rimon B. (2021). MIR172d Is Required for Floral Organ Identity and Number in Tomato. Int. J. Mol. Sci..

[95] Chung M.-Y., Nath U.K., Vrebalov J., Gapper N., Lee J.M., Lee D.-J., Kim C.K., Giovannoni J. (2020). Ectopic Expression of MiRNA172 in Tomato (*Solanum Lycopersicum*) Reveals Novel Function in Fruit Development through Regulation of an AP2 Transcription Factor. BMC Plant Biol..

[96] Karlova R., Rosin F.M., Busscher-Lange J., Parapunova V., Do P.T., Fernie A.R., Fraser P.D., Baxter C., Angenent G.C., de Maagd R.A. (2011). Transcriptome and Metabolite Profiling Show That APETALA2a Is a Major Regulator of Tomato Fruit Ripening. Plant Cell.

[97] Chung M.-Y., Vrebalov J., Alba R., Lee J., McQuinn R., Chung J.-D., Klein P., Giovannoni J. (2010). A Tomato (Solanum Lycopersicum) APETALA2/ERF Gene, SlAP2a, Is a Negative Regulator of Fruit Ripening: SlAP2a,a Negative Regulator of Tomato Fruit Ripening. Plant J..

[98] Wang R., Tavano E.C.d.R., Lammers M., Martinelli A.P., Angenent G.C., de Maagd R.A. (2019). Re-Evaluation of Transcription Factor Function in Tomato Fruit Development and Ripening with CRISPR/Cas9-Mutagenesis. Sci. Rep..

[99] Takei K., Yamaya T., Sakakibara H. (2004). Arabidopsis CYP735A1 and CYP735A2 Encode Cytokinin Hydroxylases That Catalyze the Biosynthesis of Trans-Zeatin. J. Biol. Chem..

[100] Matsuo S., Kikuchi K., Fukuda M., Honda I., Imanishi S. (2012). Roles and Regulation of Cytokinins in Tomato Fruit Development. J. Exp. Bot..

[101] Zhang Y., Yin S., Tu Y., Mei H., Yang Y. (2020). A Novel MicroRNA, SlymiR208, Promotes Leaf Senescence via Regulating Cytokinin Biosynthesis in Tomato. Physiol. Plant..

[102] Ori N., Cohen A.R., Etzioni A., Brand A., Yanai O., Shleizer S., Menda N., Amsellem Z., Efroni I., Pekker I. (2007). Regulation of LANCEOLATE by MiR319 Is Required for Compound-Leaf Development in Tomato. Nat. Genet..

[103] Zhao W., Li Z., Fan J., Hu C., Yang R., Qi X., Chen H., Zhao F., Wang S. (2015). Identification of Jasmonic Acid-Associated MicroRNAs and Characterization of the Regulatory Roles of the MiR319/TCP4 Module under Root-Knot Nematode Stress in Tomato. J. Exp. Bot..

[104] Wu F., Qi J., Meng X., Jin W. (2020). MiR319c Acts as a Positive Regulator of Tomato against Botrytis Cinerea Infection by Targeting TCP29. Plant Sci..

[105] Etemadi M., Gutjahr C., Couzigou J.-M., Zouine M., Lauressergues D., Timmers A., Audran C., Bouzayen M., Bécard G., Combier J.-P. (2014). Auxin Perception Is Required for Arbuscule Development in Arbuscular Mycorrhizal Symbiosis. Plant Physiol..

[106] Zhang Y.-Y., Hong Y.-H., Liu Y.-R., Cui J., Luan Y.-S. (2021). Function Identification of MiR394 in Tomato Resistance to Phytophthora Infestans. Plant Cell Rep..

[107] Fracasso A., Vallino M., Staropoli A., Vinale F., Amaducci S., Carra A. (2021). Increased Water Use Efficiency in MiR396-Downregulated Tomato Plants. Plant Sci..

[108] Cao D., Wang J., Ju Z., Liu Q., Li S., Tian H., Fu D., Zhu H., Luo Y., Zhu B. (2016). Regulations on Growth and Development in Tomato Cotyledon, Flower and Fruit via Destruction of MiR396 with Short Tandem Target Mimic. Plant Sci..

[109] Liebsch D., Palatnik J.F. (2020). MicroRNA MiR396, GRF Transcription Factors and GIF Co-Regulators: A Conserved Plant Growth Regulatory Module with Potential for Breeding and Biotechnology. Curr. Opin. Plant Biol..

[110] He Y., Zhou J., Hu Y., Fang C., Yu Y., Yang J., Zhu B., Ruan Y.-L., Zhu Z. (2021). Overexpression of Sly-MiR398b Increased Salt Sensitivity Likely via Regulating Antioxidant System and Photosynthesis in Tomato. Environ. Exp. Bot..

[111] Zhang C., Xian Z., Huang W., Li Z. (2015). Evidence for the Biological Function of MiR403 in Tomato Development. Sci. Hortic..

[112] Shivaprasad P.V., Chen H.-M., Patel K., Bond D.M., Santos B.A.C.M., Baulcombe D.C. (2012). A MicroRNA Superfamily Regulates Nucleotide Binding Site-Leucine-Rich Repeats and Other MRNAs. Plant Cell.

[113] Canto-Pastor A., Santos B.A.M.C., Valli A.A., Summers W., Schornack S., Baulcombe D.C. (2019). Enhanced Resistance to Bacterial and Oomycete Pathogens by Short Tandem Target Mimic RNAs in Tomato. Proc. Natl. Acad. Sci. USA.

[114] Hong Y.-H., Meng J., He X.-L., Zhang Y.-Y., Luan Y.-S. (2019). Overexpression of MiR482c in Tomato Induces Enhanced Susceptibility to Late Blight. Cells.

[115] Gao Y., Li S.-J., Zhang S.-W., Feng T., Zhang Z.-Y., Luo S.-J., Mao H.-Y., Borkovich K.A., Ouyang S.-Q. (2021). SlymiR482e-3p Mediates Tomato Wilt Disease by Modulating Ethylene Response Pathway. Plant Biotechnol. J..

[116] Mohorianu I., Schwach F., Jing R., Lopez-Gomollon S., Moxon S., Szittya G., Sorefan K., Moulton V., Dalmay T. (2011). Profiling of Short RNAs during Fleshy Fruit Development Reveals Stage-Specific SRNAome Expression Patterns: Time Course Study of Short RNAs during Fruit Development. Plant J..

[117] Jia X., Shen J., Liu H., Li F., Ding N., Gao C., Pattanaik S., Patra B., Li R., Yuan L. (2015). Small Tandem Target Mimic-Mediated Blockage of MicroRNA858 Induces Anthocyanin Accumulation in Tomato. Planta.

[118] Chen L., Meng J., He X.L., Zhang M., Luan Y.S. (2019). Solanum Lycopersicum MicroRNA1916 Targets Multiple Target Genes and Negatively Regulates the Immune Response in Tomato. Plant Cell Environ..

[119] Wang Y., Zou W., Xiao Y., Cheng L., Liu Y., Gao S., Shi Z., Jiang Y., Qi M., Xu T. (2018). MicroRNA1917 Targets CTR4 Splice Variants to Regulate Ethylene Responses in Tomato. J. Exp. Bot..

[120] Wang Y., Itaya A., Zhong X., Wu Y., Zhang J., van der Knaap E., Olmstead R., Qi Y., Ding B. (2011). Function and Evolution of a MicroRNA That Regulates a Ca2+-ATPase and Triggers the Formation of Phased Small Interfering RNAs in Tomato Reproductive Growth. Plant Cell.

