# Supplementary figures and images for: Tomato MicroRNAs and Their Functions
Source: Int J Mol Sci. 2022 Oct 9;23(19):11979. doi: 10.3390/ijms231911979 (PMC9569937; doi:10.3390/ijms231911979)

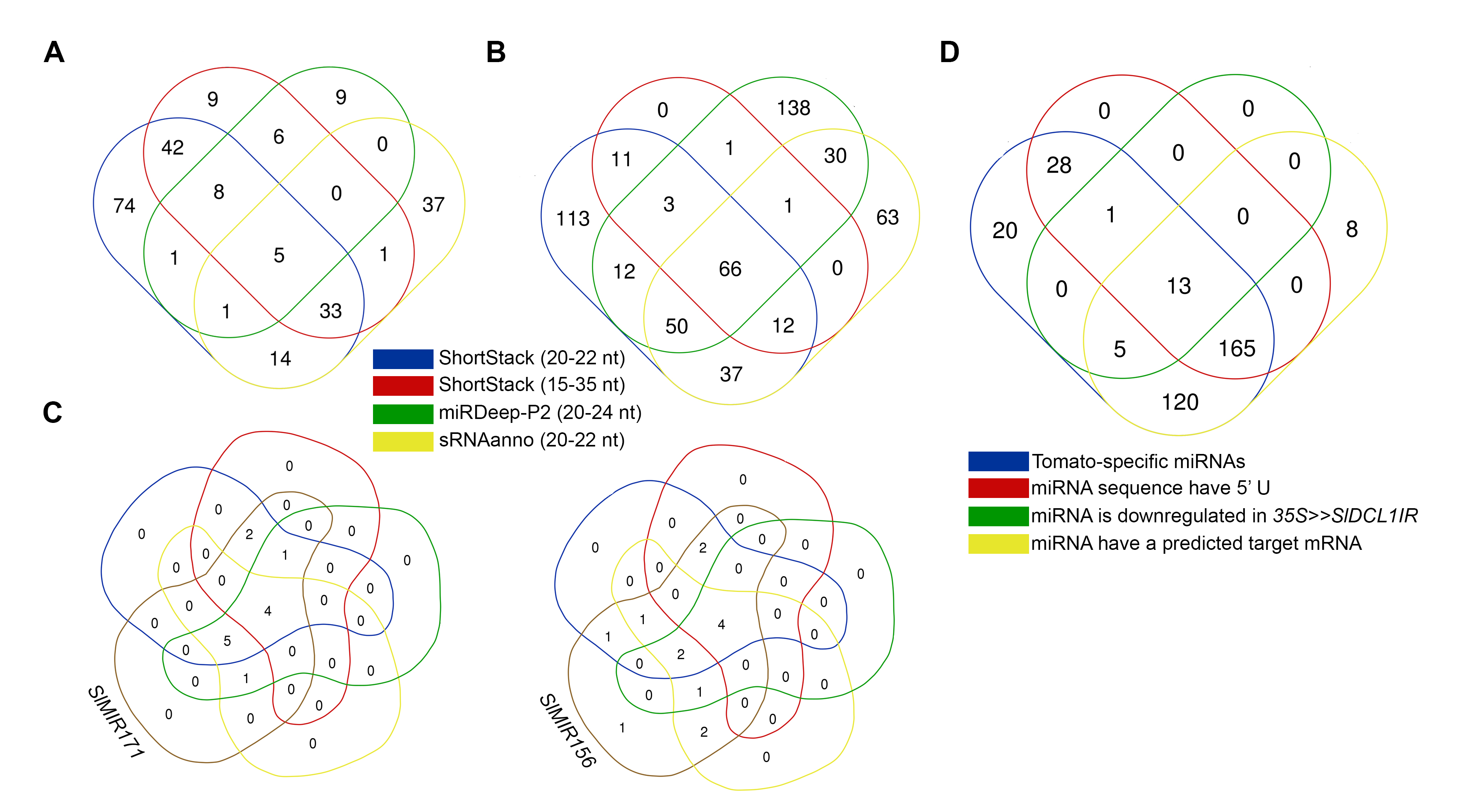

Supplement: Supplementary file 1 [file ijms-23-11979-s001.zip › Figure S1.jpg]
